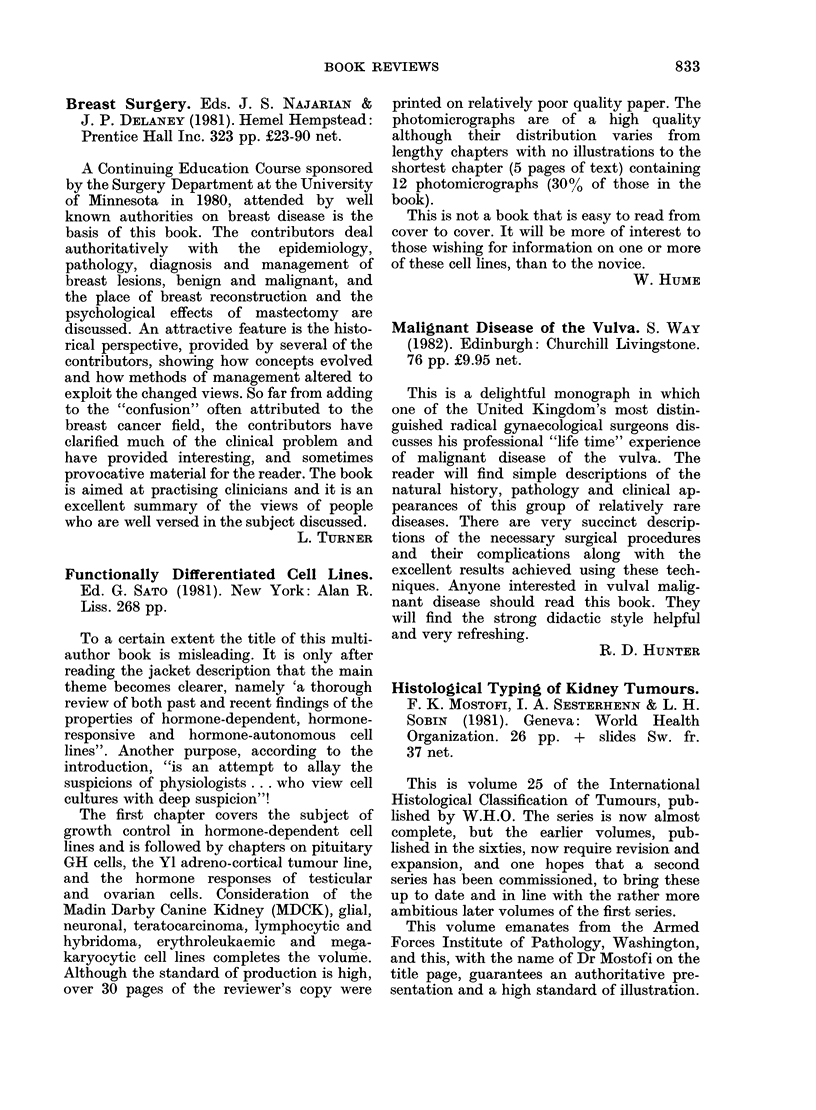# Malignant Disease of the Vulva

**Published:** 1982-11

**Authors:** R. D. Hunter


**Malignant Disease of the Vulva.** S. WAY (1982). Edinburgh: Churchill Livingstone. 76 pp. £9.95 net.

This is a delightful monograph in which one of the United Kingdom's most distinguished radical gynaecological surgeons discusses his professional "life time" experience of malignant disease of the vulva. The reader will find simple descriptions of the natural history, pathology and clinical appearances of this group of relatively rare diseases. There are very succinct descriptions of the necessary surgical procedures and their complications along with the excellent results achieved using these techniques. Anyone interested in vulval malignant disease should read this book. They will find the strong didactic style helpful and very refreshing.

R. D. HUNTER